# Design Strategies Based on Electronic Interactions for Effective Catalysts in Lithium–Sulfur Batteries

**DOI:** 10.1002/anie.202425037

**Published:** 2025-05-10

**Authors:** Donghyeok Son, Cheol‐Young Park, Jinuk Kim, Won‐Gwang Lim, Seoa Kim, Jinwoo Lee

**Affiliations:** ^1^ Department of Chemical and Biomolecular Engineering Korea Advanced Institute of Science and Technology (KAIST) 291 Daehak‐Ro, Yuseong‐Gu Daejeon 34141 South Korea; ^2^ Energy and Environment Directorate Pacific Northwest National Laboratory (PNNL) Richland WA 99354 USA

**Keywords:** Catalyst, Design principle, Electronic property, Electronic structure, Lithium–sulfur batteries

## Abstract

Lithium–sulfur batteries (LSBs) are considered promising next‐generation batteries due to their high energy density (>500 W h kg^−1^). However, LSBs exhibit an unsatisfactory energy density (<400 W h kg^−1^) and cycle life (<300 cycles) because of the shuttle effect caused by soluble lithium polysulfide (LiPS) intermediates and the sluggish conversion reaction kinetics caused by insulating sulfur (S_8_) and lithium sulfide (Li_2_S). Although various types of catalysts, including metal‐based compounds to single‐atom catalysts, have been reported to address these issues, most catalysts exhibited limited catalytic activity under practical lean electrolyte conditions (<5 µL mg^−1^). A comprehensive understanding of the synthetic strategy and catalytic mechanism of catalysts is essential for their design, but understanding the electronic effects of the catalysts and LiPS is more important. Furthermore, the electronic design of these catalysts is not well understood. In this review, we introduce the catalytic mechanisms in LSBs and discuss catalyst design strategies in terms of electronic effects on the interactions between reactants and catalysts, with a primary focus on heterogeneous catalytic systems. We additionally consider how the electronic property of homogeneous systems, particularly redox mediators, affects catalytic behavior under lean electrolyte conditions and propose future research directions for catalyst development in LSBs.

## Graphical abstract

Design of effective bidirectional electrocatalyst focused on electronic interactions in lithium–sulfur batteries. Development of effective bidirectional catalyst is essential for high‐energy density and long‐life lithium–sulfur batteries. The catalytic activity of catalyst is directly related to the degree of the interaction between the catalysts and adsorbates. In the review, we introduce the design strategy for catalysts with high catalytic activity focused on the electronic structure and properties of catalysts and reactants.

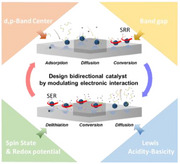

## 1 Introduction

As the demand for high‐energy‐density batteries increases, there is growing research interest in next‐generation batteries that could potentially replace lithium‐ion batteries (LIBs),^[^ ^1^, ^2^, ^3^, ^4^ ^]^ which have a limited energy density (<350 W h kg^−1^) due to the intercalation chemistry of the electrodes.^[^ ^5^, ^6^ ^]^ Among these, lithium–sulfur batteries (LSBs) are emerging as promising candidates. LSBs are expected to exhibit high energy density (>500 W h kg^−1^)^[^ ^7^ ^]^ by adopting sulfur as a cathode, which exhibits a high theoretical specific capacity (1672 mA h g^−1^) with conversion chemistry,^[^ ^8^ ^]^ and lithium (Li) as an anode, which has a high theoretical specific capacity (3860 mA h g^−1^) and low redox potential (−3.04 V vs. SHE).^[^ ^9^ ^]^ However, the commercialization of LSBs is hindered by several drawbacks: 1) the shuttle effect by diffusion of soluble lithium polysulfide intermediates (Li_2_S*_x_*, *x* = 4–8) between the cathode and anode, which reduces the coulombic efficiency and cycle stability^[^ ^10^, ^11^, ^12^ ^]^ and 2) sluggish reaction kinetics from the insulating properties of sulfur and lithium sulfide (Li_2_S), which results in poor rate performance and low energy density.^[^ ^13^ ^]^

Extensive research has been conducted over the past decades to address these challenges. Nazar's group reported CMK‐3,^[^ ^14^ ^]^ a highly ordered mesoporous carbon, as an electrically conductive host material that can prevent the shuttle effect and improve the conversion reaction kinetics through the physical entrapment of soluble lithium polysulfides (LiPSs) and facile electrolyte infiltration. However, the physical adsorption of LiPSs based on van der Waals forces is insufficient to effectively suppress the shuttle effect, and nonpolar carbon materials exhibit poor electrolyte wettability, resulting in low cycle stability and reversible capacity.^[^ ^15^, ^16^ ^]^ To overcome these drawbacks, substantial research has been carried out on polar and conductive host materials that can strongly entrap soluble LiPSs via chemical adsorption and enhance the conversion reaction kinetics by enhancing the electrolyte wettability.^[^ ^17^ ^]^ However, strategies based on blocking the dissolution of soluble LiPS have demonstrated insufficient suppression of the shuttle effect and conversion reaction kinetics under low electrolyte‐to‐sulfur (*E*/*S*) ratio conditions (*E*/*S* ratio < 5 µL mg^−1^) for practical LSBs when the high concentration gradient of LiPSs and the viscosity of the electrolyte exacerbate the shuttle effect and conversion reaction kinetics, respectively.^[^ ^18^, ^19^ ^]^

Therefore, a significant amount of research has been conducted on catalyzing sluggish sulfur conversion reactions with catalytic materials, as they can simultaneously address the issues of poor rate capability and the shuttle effect by reducing the residence time of soluble LiPSs.^[^ ^20^ ^]^ Although various types of catalytic materials (single‐atom catalysts (SACs),^[^ ^21^, ^22^, ^23^ ^]^ transition metal compounds (TMCs),^[^ ^24^, ^25^, ^26^, ^27^ ^]^ and nonmetallic materials^[^ ^28^, ^29^ ^]^) have been introduced to facilitate the sulfur conversion reaction, resulting in significant improvements in reversible capacity and cycle stability, there is still a lack of research on the underlying catalytic mechanisms and methods for catalyst design. Furthermore, although operation under lean electrolyte conditions is essential for high energy density LSBs, most recently reported catalysts have been subjected to electrochemical analysis and performance evaluation under flooded electrolyte conditions (*E*/*S* > 5 µL mg^−1^, Table 1).

**Table 1:** The electrochemical performance of catalysts reported in LSBs.

Catalytic materials	Initial capacities (mA h g^−1^)	Retained capacities (mA h g^−1^)	*E*/*S* ratio (µL mg^−1^)	Sulfur loading (mg cm^−2^)	C‐rate	Cycle number
NiCoP@CoP^[^ ^30^ ^]^	1063	257	15	1.2	1	1000
MoC–MoSe_2_^[^ ^31^ ^]^	666.7	777.8	9	5.4	0.5	50
CoNC@ZnNC^[^ ^32^ ^]^	972	800	6	4.2	0.2	160
CNTs@COF–SO_3_H^[^ ^33^ ^]^	600	576.5	8.69	6.9	0.1	150
NiCoNC^[^ ^34^ ^]^	800	494.2	8	4.5	0.2	200
ZIF@CNTs/Co@CNFs^[^ ^35^ ^]^	1175	875	5	4	0.1	100
Co─N_3_Cl_1_^[^ ^36^ ^]^	933.3	800.8	5.4	7.5	0.1	50
FeN_2_C_2_–SO*_x_*–N^[^ ^37^ ^]^	686.7	673.0	5.1	5.17	0.2	40
*o*‐CoTe_2_^[^ ^38^ ^]^	1015.6	937.5	5.4	6.4	0.1	50
NCF@MoSe_2_,MoSe_2_/MoP^[^ ^39^ ^]^	872.7	909.1	7	5.5	0.2	50
Co_0.125_Zn_0.875_Se^[^ ^40^ ^]^	893.9	681.8	6	6.6	0.1	80
NiS_2_/NiSe_2_@NC^[^ ^41^ ^]^	900	720.1	7.1	6.2	0.1	300
Co_0.75_Fe_0.25_P@C^[^ ^42^ ^]^	905.4	807.1	10	5.6	0.1	52
CoSA–N_3_PS^[^ ^43^ ^]^	800	660	8	6	0.2	100
Co@NCNT–MoSe_2_^[^ ^44^ ^]^	1048	760	7.5	5	0.2	50
FeNC^[^ ^45^ ^]^	850	720	6	4	0.05	85
Co/CoV_2_O_6_/NC^[^ ^46^ ^]^	789.3	558.4	16	1.1	0.2	100
NbB_2_/rGO/PP^[^ ^47^ ^]^	566.6	637.4	8	7.06	0.1	40
PCC@CoSe^[^ ^48^ ^]^	812	726	7	4.68	0.1	70
FeCo–NC^[^ ^49^ ^]^	1064.2	931.3	5.5	6.7	0.1	50
C‐doped TiO_2_ *_–x_* ^[^ ^50^ ^]^	1149	769	12	2.2	0.2	100
P‐MoTe_2_/CC^[^ ^51^ ^]^	957.5	662.7	14.8	2	1	1000
SAMn@NSC^[^ ^52^ ^]^	995	628.9	15	1	0.5	300
FeNC–EEB‐1^[^ ^21^ ^]^	∼1300	∼1050	10	2	0.2	200
MoS_2_/rGO‐5.6^[^ ^53^ ^]^	1265	901	30	1.5	0.2	200
Co_0.125_Zn_0.875_S^[^ ^54^ ^]^	1163.2	856.1	10	2	1	200
Co_9_S_8_@MoS_2_^[^ ^55^ ^]^	1117	794	17.69	3	1	400
SC–TiO_2_–Hal^[^ ^56^ ^]^	1199.1	1045.6	10	–	0.2	200
V‐Co_3_O_4_NS^[^ ^57^ ^]^	1093.5	994.1	15	1–2	1	500
CNF/CoS*_x_* ^[^ ^58^ ^]^	1020	315	25	1–1.2	2	8150
Ni_6_(BTB)_4_(BP)_3_^[^ ^59^ ^]^	686	611	–	–	0.2	100
WO*_x_*‐PANi/rGO^[^ ^60^ ^]^	∼1050	683.1	40	0.8–1.0	0.3	300
Sb_2_Se_3_ *_–x_*/rGO^[^ ^61^ ^]^	1387	1125	15	1.8	0.1	100
CoNi–MOF^[^ ^62^ ^]^	963	790	20	1.5	1	500
ZnS_1_ *_–x_*–CC^[^ ^63^ ^]^	659	524	15	1.7	1	500

Chen et al. reported that the kinetic overpotential (*η*_act_) increases most significantly as the *E*/*S* ratio decreases in LSBs through galvanostatic intermittent titration technique‐electrochemical impedance spectroscopy (GITT‐EIS) analysis.^[^ ^19^ ^]^ Therefore, under lean electrolyte conditions, introducing electrocatalysts capable of mitigating kinetic overpotential could be a promising strategy for achieving high‐performance LSBs. However, in lean electrolyte environments, the high viscosity of the electrolyte reduces accessibility to the triple‐phase interface (catalyst/conductive support/electrolyte),^[^ ^64^, ^65^ ^]^ and exacerbated LiPS clustering leads to high reaction barriers, making it difficult for the catalysts to exhibit high activity.^[^ ^66^ ^]^ Under such constrained environments, the intrinsic catalytic activity governed largely by the catalyst's electronic structure plays a decisive role in determining performance.^[^ ^21^, ^67^ ^]^ Tailoring the electronic structure of catalysts can modulate the adsorption energy of LiPS intermediates and lower the activation energy of sulfur conversion reactions, thereby improving catalytic efficiency even under lean electrolyte conditions. Therefore, a deeper understanding of the catalytic mechanisms and more intensive research on the design of catalysts with high activities are crucial for developing catalysts that perform effectively even under lean electrolyte conditions in LSBs.

Although heterogeneous catalysts have been predominantly investigated to promote sulfur conversion reactions, recent studies have also highlighted the role of homogeneous catalysts, such as redox mediators (RMs),^[^ ^68^, ^69^ ^]^ particularly under lean electrolyte conditions, where the high viscosity of the electrolyte limits ionic conductivity and mass transport, thereby hindering the effectiveness of conventional surface‐based catalysis. In addition, RMs are solvated in the electrolyte, facilitating access to the electrode surface without the limitations of triple‐phase interfaces. Depending on their redox potentials, RMs can accelerate the Li_2_S formation reactions. Moreover, based on their HOMO–LUMO energy levels, RMs can reactivate passivated species such as dead Li_2_S or cyclo‐octasulfur (S_8_) on the electrode surface. These unique properties make RMs attractive as effective catalytic components under lean electrolyte conditions.

Catalysis is a process that occurs through the interaction between the catalyst and reactants, the extent of which is closely related to the electronic distribution and structure of both the catalyst and reactants and significantly influences the kinetic activity of the catalyst.^[^ ^70^, ^71^, ^72^ ^]^ Therefore, a deep understanding of the electronic interactions between catalysts and reactants is essential for the rational design of catalysts in LSBs. In this review, we introduce the catalytic mechanisms in LSBs, discuss catalyst design strategies in terms of electronic effects, specifically from the perspective of electronic structure and properties, on the interactions between reactants and catalysts. We also propose future research directions for catalyst development in LSBs.

### 1.1 Working Principle of LSBs

**Figure 1:**
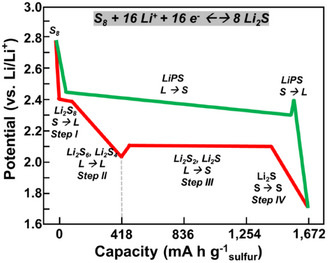
Typical voltage profile and operating principles of LSBs.

A thorough understanding of the working principles of LSBs is crucial for the tailored design of LSB catalysts. Figure 1 shows a typical voltage profile of an LSB. During charging and discharging in LSBs, a reversible conversion reaction occurs between S_8_ and Li_2_S^[^ ^73^, ^74^ ^]^ as represented by the following overall reactions:

Overall reaction in LSBs:

Discharge: S_8_ + 16 Li^+^ + 16 e^−^ → 8 Li_2_S

Sulfur reduction reaction (SRR)

Charge: 8 Li_2_S → S_8_ + 16 Li^+^ + 16 e^−^

Sulfur evolution reaction (SER)

However, the conversion between S_8_ and Li_2_S is a multistep process, involving multiple electrons and various forms of Li_2_S*_x_* (*x* = 2, 4, 6, and 8).^[^ ^75^, ^76^, ^77^ ^]^ Furthermore, it consists of not only electrochemical reactions but also chemical reactions of disproportionation and comproportionation.

During discharge, S_8_ undergoes lithiation reactions, sequentially forming various soluble LiPS intermediates (Li_2_S_8_ → Li_2_S_6_ → Li_2_S_4_) and transitioning to solid state Li_2_S_2_ and Li_2_S. The overall discharge process in LSBs can be divided into four steps in the voltage profile owing to the phase transition of the active species (Figure 1).

Discharge

Step 1: solid‐to‐liquid reaction

S_8_ + 2 Li^+^ + 2 e^−^ → Li_2_S_8_

Step 2: liquid‐to‐liquid reaction

1) 3 Li_2_S_8_ + 2 Li^+^ + 2 e^−^ → 4 Li_2_S_6_

2) 2 Li_2_S_6_ + 2 Li^+^ + 2 e^−^ → 3 Li_2_S_4_

 ⇒Li_2_S_8_ + 2 Li^+^ + 2 e^−^ → 2 Li_2_S_4_

Step 3: liquid‐to‐solid reaction

2 Li_2_S_4_ + 4 Li^+^ + 4 e^−^ → 4 Li_2_S_2_

Step 4: solid‐to‐solid reaction

4 Li_2_S_2_ + 8 Li^+^ + 8 e^−^→ 8 Li_2_S

First, S_8_ undergoes lithiation through a ring‐opening reaction, leading to its conversion to Li_2_S_8_, achieving a capacity of 209 mA h g^−1^. Second, Li_2_S_8_ is sequentially converted to Li_2_S_6_ and then to Li_2_S_4_, achieving a similar capacity of 209 mA h g^−1^. In addition, long‐chain LiPS intermediates (Li_2_S*_x_*, *x* = 4, 6, and 8) are soluble in the electrolyte, leading to the shuttle effect.^[^ ^78^, ^79^ ^]^ Third, insoluble Li_2_S_2_/Li_2_S nucleation occurs in the following steps, exhibiting a potential dip. Due to the insulating properties of Li_2_S_2_ and Li_2_S, the reaction rates of steps 3 and 4 are extremely slow and are considered the rate‐determining steps (RDS) in the SRR, exhibiting a potential well in the initial portion of the discharging voltage curve. However, since the reactions in steps 3 and 4 deliver 1252 mA h g^−1^, representing 75% of the total capacity, it is essential to promote these reactions.

Conversely, during charging, Li_2_S is first converted into Li_2_S*_x_* intermediates and eventually transformed into S_8_ through delithiation.

Charge

8 Li_2_S → S_8_ + 16 Li^+^ + 16 e^−^

Due to the insulating property of Li_2_S, the Li_2_S delithiation reaction (Li_2_S → LiS + Li^+^ + e^−^) through breakage of the Li─S bond is considered the RDS in the SER,^[^ ^80^, ^81^ ^]^ exhibiting a potential hill in the initial portion of the charging voltage curve. If the SER does not proceed effectively, the insulating Li_2_S remaining on the electrode surface hinders the catalytic activity, thereby making it difficult for the catalyst to sustain its activity throughout continuous charge–discharge cycles. In summary, the multielectron charge–discharge process of LSBs and the formation of soluble intermediates are detrimental to the electrochemistry of LSBs, and their sluggish kinetics need to be accelerated. Therefore, the energy barrier of the RDS of both the SRR and SER should be lowered for continuous catalytic performance.^[^ ^82^, ^83^ ^]^

### 1.2 Roles of Catalysts and Catalytic Mechanism in LSBs

Similar to the conventional role of catalysts, catalysts in LSBs facilitate reactions by lowering the activation barrier of the RDS without directly participating in the reaction. The introduction of a catalyst is a particularly effective strategy for improving the energy density and cycle stability of LSBs because the sluggish conversion reaction involving insulating solid‐phase active species can be resolved by lowering the activation barrier of the RDS through strong adsorption, accelerated conversion, and fast charge transfer.

**Figure 2:**
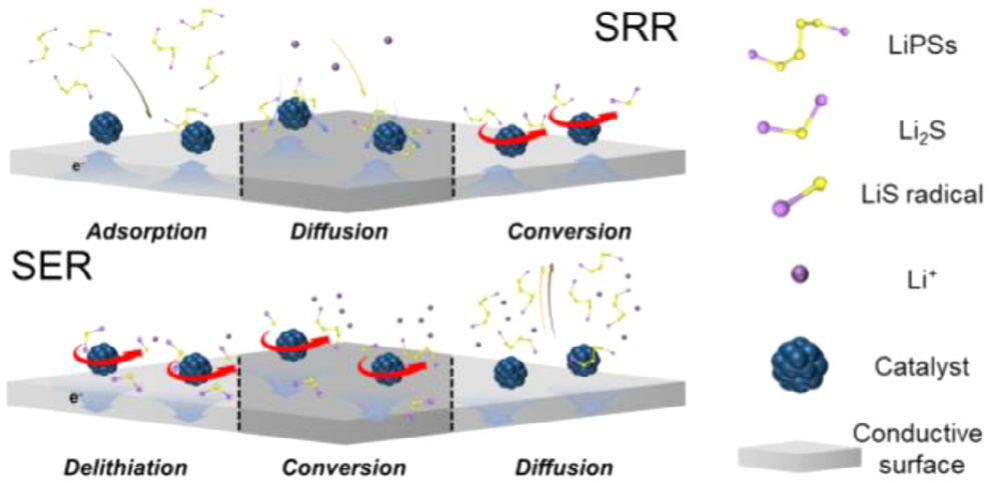
Schematic diagram of the catalysis process of SRR and SER.

Understanding the catalytic mechanism of LSBs is essential for developing effective catalysts. Facile catalysis in both the SRR and SER is essential for reversible charge–discharge cycles.^[^ ^9^, ^20^ ^]^ As shown in Figure 2, the catalytic mechanism differs between the SRR and SER. The SRR consists of three steps: adsorption, diffusion, and conversion.^[^ ^84^, ^85^, ^86^ ^]^ In the SRR, catalysis begins with the adsorption of LiPSs onto the catalyst surface. Once adsorbed, the LiPSs and Li^+^ ions diffuse toward the triple‐phase interfaces, which is the catalytically active center. Subsequently, LiPSs are converted to Li_2_S_2_/Li_2_S through reactions with the transferred electrons and diffused Li^+^ ions appear on the catalyst surface. Previously, it was believed that the stronger the adsorption of a catalyst on LiPSs, the better the battery performance in addressing the shuttling issue of soluble LiPSs. However, it is now recognized that a well‐balanced interplay among the three interdependent steps (adsorption, surface diffusion, and conversion) is essential for efficient overall catalysis of the SRR.^[^ ^87^, ^88^ ^]^ From an electronic perspective, when adsorption is too strong, the extensive orbital overlap from the strong interaction between the catalyst and LiPS facilitates efficient charge transfer, promoting the conversion process to Li_2_S_2_/Li_2_S. However, the diffusion of Li⁺ ions and LiPSs toward triple‐phase interfaces is impeded, hindering reversible charge–discharge cycles. Conversely, if the adsorption is too weak, diffusion occurs more easily, but the conversion process to Li_2_S_2_/Li_2_S is less effective because of insufficient charge transfer by the small orbital overlap owing to the weak interaction between the LiPSs and the catalyst.

The SER consists of three steps: delithiation, conversion, and diffusion (Figure 2).^[^ ^84^ ^]^ In the SER, catalysis begins with the delithiation of the Li─S bond in the Li_2_S deposits on the catalyst surface. The delithiation of Li_2_S has a high activation barrier because of its insulating nature and insufficient contact between solid‐phase Li_2_S and catalysts, which makes it the RDS of the SER. After the delithiation of Li_2_S, it is converted into long‐chain LiPSs through multielectron reactions. Ensuring the electrical conductivity of the catalyst is crucial for facilitating the conversion process.^[^ ^89^, ^90^ ^]^ After the conversion step, Li^+^ ions and LiPSs diffuse into the bulk electrolyte. The high viscosity of the electrolyte caused by the soluble LiPSs hinders the diffusion of Li⁺ ions and LiPSs within the electrolyte, adversely affecting the rate performance of LSBs. Similar to the SRR, the interaction between various forms of sulfur‐containing active materials and the catalyst in the SER significantly influences catalysis. When the interaction between the reactants and catalyst is too strong, the Li─S bonds within Li_2_S weaken, facilitating the delithiation and conversion steps.^[^ ^71^, ^81^ ^]^ However, interactions between the sulfur‐containing active materials and the catalyst that are too strong hinder the diffusion of Li⁺ ions and LiPSs into the bulk electrolyte.^[^ ^85^ ^]^ Conversely, if the interaction is too weak, diffusion occurs readily, but the delithiation and conversion steps are hindered.

In summary, designing bidirectional catalytic materials that are effective for both the SRR and SER requires tuning the interaction between sulfur‐containing active materials and the catalyst to ensure facile catalysis by balancing the steps in both the SRR (adsorption–diffusion–conversion) and SER (delithiation–conversion–diffusion). Therefore, understanding the electronic structures and properties of the reactants and catalysts can be used to fine‐tune the interactions to facilitate the SRR and SER, which will enhance the energy density and cycle stability of LSBs. In contrast, the preceding discussion addresses the general catalytic mechanisms in LSBs. However, under lean electrolyte conditions, the significantly increased concentration of LiPSs can lead to precipitation^[^ ^91^ ^]^ and gelation,^[^ ^92^ ^]^ which may fundamentally alter the catalytic pathways. Despite this, the impact of lean electrolyte environments on the catalytic mechanisms remains insufficiently explored. Therefore, an in‐depth investigation into the catalytic behavior under lean electrolyte conditions is essential, as it would provide critical guidance for the rational design of catalysts optimized for high‐performance LSBs.

Although this section focuses on the surface‐mediated catalytic mechanisms of heterogeneous catalysts, understanding the solution‐mediated electron transfer mechanisms of RMs, which are solvated in the electrolyte, is also important for promoting sulfur conversion reactions. While their fundamental mechanisms differ from those of heterogeneous catalysts, their roles will be discussed in the following sections in the context of catalyst design strategies based on electronic interactions.

## 2 Design Principles of Catalyst in LSBs

To ensure effective catalytic activity in both the SRR and SER, it is crucial to optimize the interactions between the reactants and catalysts. For heterogeneous catalysts, these interactions are governed by electronic structure parameters such as the d‐band, p‐band centers, and band gap, as well as electronic properties, including spin states and Lewis acidity/basicity, of both the reactants and catalysts. In contrast, for homogeneous catalysts such as RMs, the interactions are primarily influenced by their redox potentials as electronic properties. In the following content, the design strategies for effective bidirectional catalysts based on the modulation of electronic interaction between the catalyst and reactant will be discussed.

### 2.1 Electronic Structure

Interactions between the heterogeneous catalysts and reactants vary according to their electronic structures, which can be effectively represented by their density of states (DOS). DOS refers to the number of available states per unit energy interval that can be occupied by electrons. Specifically, during these reactions, electrons in the valence orbitals hybridize with the adsorbate, making the projected DOS (pDOS) of the outermost electrons more relevant than that of the entire set of orbitals. The electronic state of a catalyst relative to its Fermi level (*E*_F_) is a key factor in determining the interactions between adsorbates. Therefore, the position of the d,p‐band centers relative to the *E*_F_, as revealed through the DOS, serves as an effective indicator of the interactions between the reactants and catalysts. High‐activity catalysts can be designed by appropriately adjusting their band centers.

#### 2.1.1 d‐Band Center

Transition metal compounds (TMCs) are widely used as heterogeneous catalysts for LSBs because of their strong interactions with LiPS and the versatility of their electronic structures.^[^ ^93^, ^94^, ^95^ ^]^ Although electrons occupy the d‐orbitals of cationic metals, and the p‐orbitals of anions can participate in the reduction and oxidation of LiPS, the d‐orbitals are considered to represent the interactions between the catalysts and LiPS. This is because of the higher influence of the d‐orbitals on the narrowness of the electron distribution and their higher electron energy level compared to p‐orbitals. Therefore, understanding d‐orbitals and strategies to modify their electronic structures are essential for developing advanced catalysts for LSBs.^[^ ^96^ ^]^ The d‐band center theory explains the influence of the electronic structure of a catalyst on the binding energy of adsorbates to TM‐based catalysts.^[^ ^97^ ^]^ Specifically, the binding energy varies with the position of the d‐band center relative to the E_F_, affecting the strength of the interactions between the adsorbate and the TM. This relationship aids in understanding the catalytic activity and selectivity of various catalysts. When LiPS is adsorbed onto the catalysts, the d‐orbitals of the catalysts and the p‐orbitals of sulfur in LiPS hybridize to form bonding and antibonding orbitals. Specifically, if the d‐band center of TMs shifts upward, the number of electrons occupying antibonding orbitals decreases, resulting in high adsorption strength with LiPS. Conversely, if the d‐band center is low, more electrons contribute to the antibonding states, thereby weakening the bonds.^[^ ^98^ ^]^ Therefore, it is crucial to design catalysts with optimized electronic structures to achieve balanced adsorption strength toward LiPS and improve catalytic activity.

However, in the early stages of catalyst development for LSBs, there was a lack of understanding regarding the catalytic mechanisms. Therefore, there was a focus on adjusting the d‐band center of the catalytic materials to be near the *E*_F_ to increase the binding energy and address the shuttle effect. Various strategies have been implemented to tune the d‐band center, including compositional engineering (doping,^[^ ^99^, ^100^ ^]^ alloying,^[^ ^101^, ^102^ ^]^ and defect engineering^[^ ^63^, ^103^ ^]^) and structural engineering (strain effect^[^ ^104^, ^105^ ^]^ and heterostructure^[^ ^55^, ^106^ ^]^).

**Figure 3:**
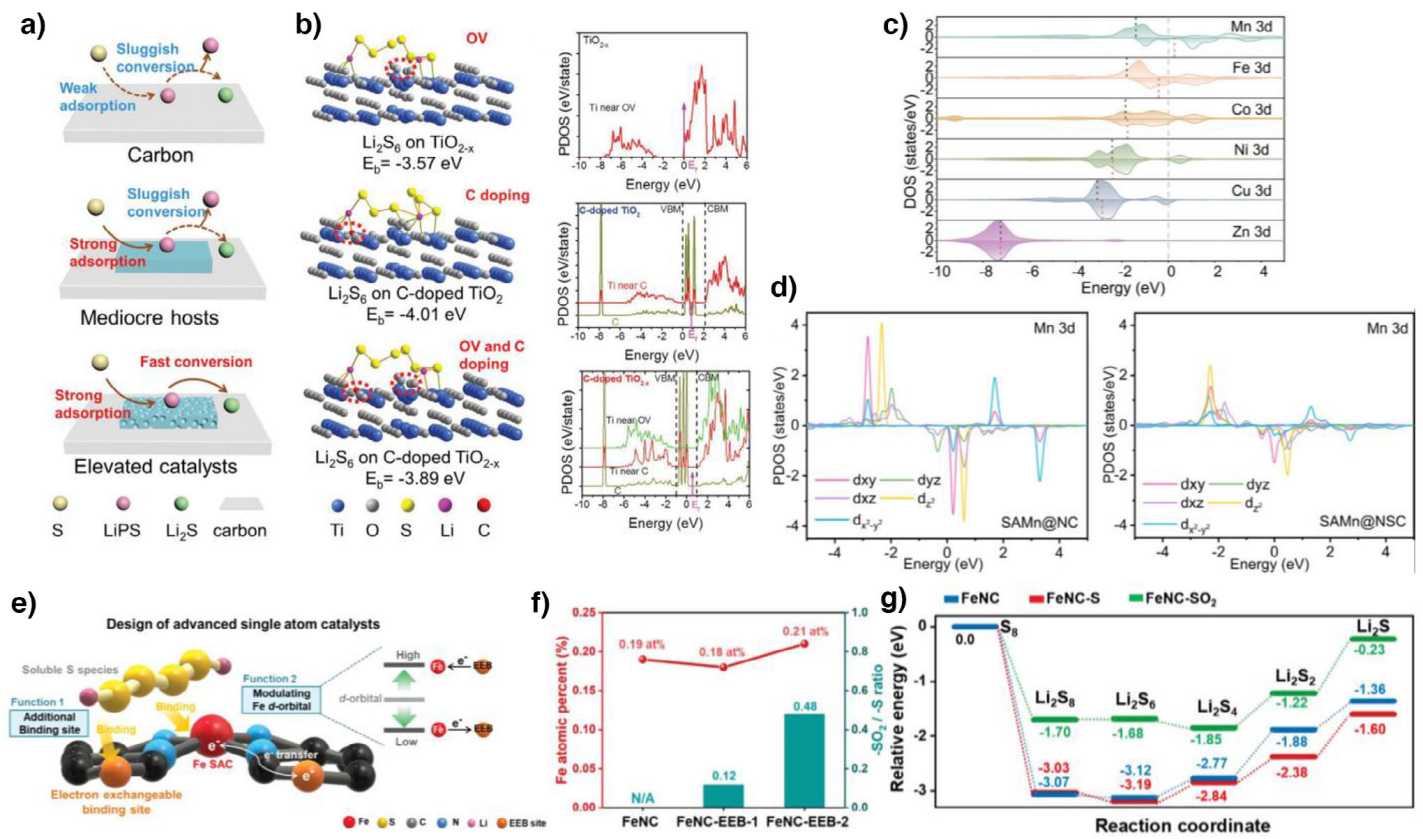
a) Improved catalysis through simultaneous defect and heterojunction engineering. b) Li_2_S_6_ binding energies and pDOS of TiO_2–_ *_x_*, C‐doped TiO_2_, and C‐doped TiO_2–_ *_x_*. Copyright 2019 Wiley‐VCH. c) d‐Band center modulation of SAMn@NSC by heteroatom coordination and its effects on d‐band splitting. d) pDOS of Mn 3d for SAMn@NC and SAMn@NSC. Copyright 2024 Elsevier. e) Schematic representation of the modulation of the d‐band center of Fe–N–C through electron‐exchangeable binding sites. f) ─SO_2_/─S ratios of FeNC, FeNC‐EEB‐1, and FeNC‐EEB‐2. g) Energy diagrams for FeNC with different local environments. Copyright 2023 Wiley‐VCH.

Wang et al. synthesized defective TiO_2_ on carbon nanotubes (C‐doped TiO_2_ *_–x_*) to improve the catalytic activity (Figure 3a).^[^ ^50^ ^]^ Through electronic structure reorganization induced by oxygen defects and carbon doping, the d‐band center of C‐doped TiO_2_ *_–x_* was upshifted to the *E*_F_, resulting in an improved binding energy and a reduced band gap (Figure 3b). Thus, C‐doped TiO_2_ *_–x_* exhibited high stability over 500 cycles at 1 C and rate performance at 5 C owing to the improved catalytic activity of TiO_2_.

Single‐atom catalysts (SACs) are widely used as catalysts for LSBs owing to high atom utilization efficiency (∼100%).^[^ ^107^, ^108^ ^]^ Additionally, SACs are attractive because of their versatility, as they allow for facile electronic structure engineering^[^ ^109^ ^]^ through both control of the metal atom center and the surrounding environment.^[^ ^110^, ^111^ ^]^ Li et al. fabricated six different sulfur‐doped SACs (TMs‐N_3_S, Mn, Fe, Co, Ni, Cu, and Zn) and investigated their effects on the pDOS (Figure 3c).^[^ ^52^ ^]^ Not only was the d‐orbital affected by the central metal atoms, but the variation in the coordination environment from TMs‐N_4_ to TMs‐N_3_S also changed the configuration of the d‐orbital by elevating the energy levels of the d*_z_*_2_ and d*_xz_* orbitals (Figure 3d). According to their results, Mn (SAMn@NSC) maintained a high capacity of 4.4 mA h cm^−2^ after 75 cycles at 0.1 C under a high sulfur loading of 6.0 mg cm^−2^ and lean electrolyte of 4.5 µL mg^−1^ by the highest d‐band center of Mn–N_3_S. This implies the strongest adsorption to LiPS among the other catalysts. Doping with heteroatoms or functional groups is an effective strategy for changing the electronic structure of SACs because different electrophilicities induce a change in the state of electron filling in the d‐orbital. Lim et al. investigated the effects of electron‐exchangeable binding (EEB) sites, electron donors (─S), and electron acceptors (─SO_2_) on Fe–N–C catalysts (Figure 3e).^[^ ^21^ ^]^ By varying the precursor, they adjusted the ratio of electron donors to acceptors around the Fe–N–C, ultimately controlling the electronic structure. With an optimally controlled ─S/─SO_2_ ratio of 0.12 when more electron donors were located near the Fe–N–C catalysts (FeNC‐EEB‐1), the d‐orbital level of the Fe atom shifted upward (Figure 3f). Owing to the facile adsorption, diffusion, and conversion reactions through d‐band center engineering, the FeNC‐EEB‐1 cathode could adsorb LiPSs and reduce dissolution while effectively lowering the energy barrier of the RDS (Figure 3g). Therefore, FeNC‐EEB‐1 exhibited high catalytic activity under a high sulfur loading of 8.4 mg cm^−2^ and a low *E*/*S* ratio of 3.0 µL mg^−1^.

**Figure 4:**
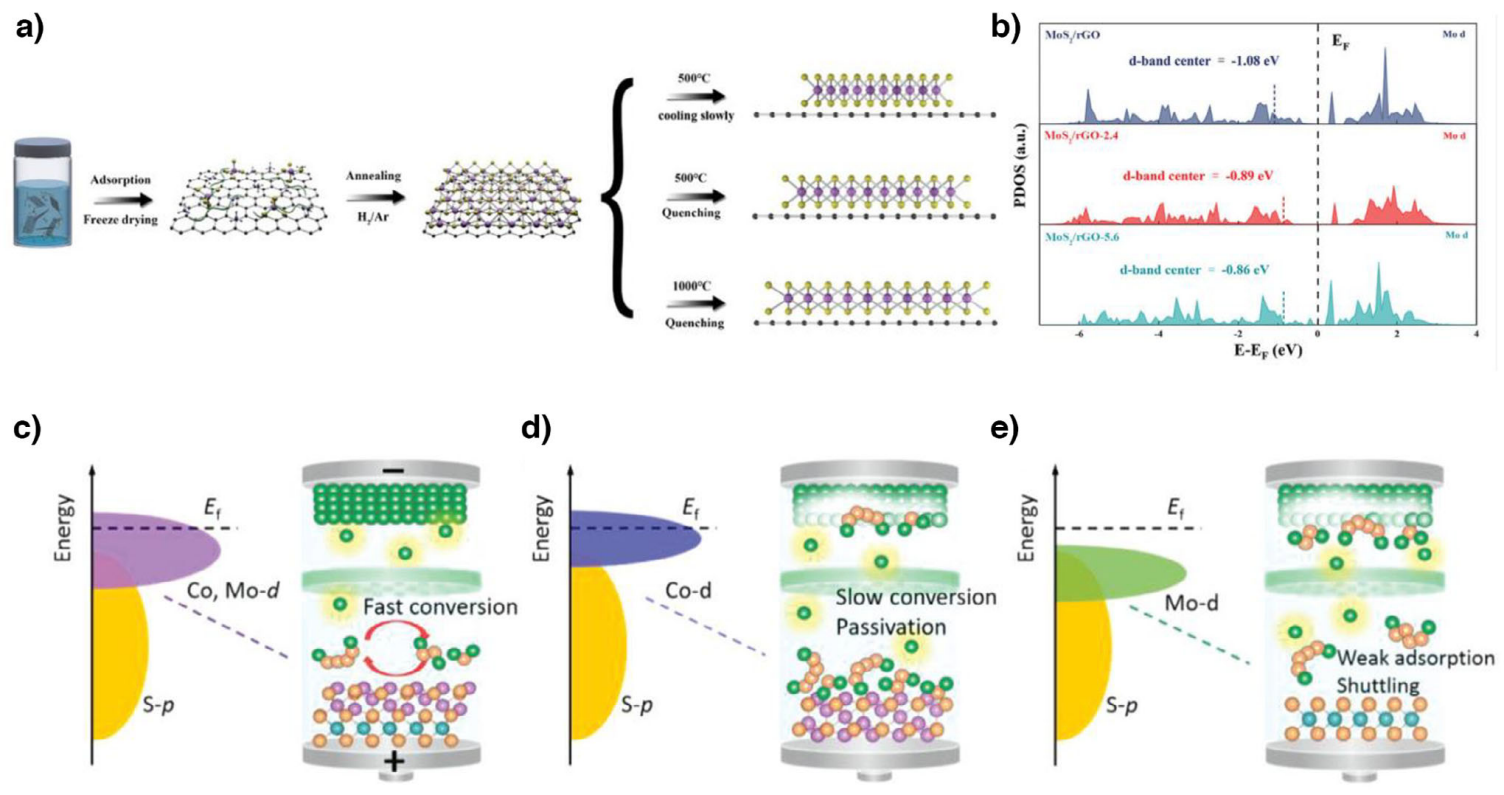
a) Description of tensile‐strained MoS_2_/rGO by the quenching strategy. b) Elevated d‐band center of MoS_2_/rGO‐2.4 and MoS_2_/rGO‐5.6 through tensile strain. Copyright 2024 Wiley‐VCH. c) Optimized d‐band center of Co_9_S_8_@MoS_2_ between those of d) Co_9_S_8_ and e) MoS_2_. Copyright 2022 Wiley‐VCH.

Structural engineering involving the application of strain to catalysts can induce changes in their electronic structures.^[^ ^100^, ^112^ ^]^ Zhao et al. investigated the pure effect of strain on the catalytic activity based on the electronic structure by applying strain to MoS_2_ through heat treatment and quenching processes.^[^ ^53^ ^]^ The MoS_2_ did not fully contract during rapid cooling, leading to tensile stress (Figure 4a). By comparing the lattice spacing, they found that quenching at 500 and 1000 °C resulted in 2.4% (MoS_2_/rGO‐2.4) and 5.6% (MoS_2_/rGO‐5.6) tensile strain, respectively. In Figure 4b, density functional theory (DFT) calculations showed that the d‐band center of MoS_2_/rGO‐5.6 shifted upward to −0.86 eV, compared to MoS_2_/rGO (−1.08 eV), reducing the electron occupation in the antibonding states and increasing the adsorption energy. Furthermore, the elongated Mo─S bond in MoS_2_ due to tensile stress weakened the Li─S bond in Li_2_S and S─S bond in Li_2_S_2_, lowering the delithiation barrier of Li_2_S. Similar results were obtained by different lattice mismatch‐induced strained MoS_2_. Zhang et al. synthesized the core‐shell structured nanoparticles on carbon nanofiber, CNF/s‐MoS_2_(Co_9_S_8_), in which thin MoS_2_ wrapped the core‐Co_9_S_8_.^[^ ^104^ ^]^ The larger lattice parameter of Co_9_S_8_ distorted the monolayer of MoS_2_ grown on Co_9_S_8_. They figured out that MoS_2_ grown on Co_9_S_8_ has 2.1% tensile strain compared to pristine MoS_2_. Due to electronic structure modulation induced by tensile strain, sulfur cathode with CNF/s‐MoS_2_(Co_9_S_8_) delivered high capacity of 1289 mA h g^−1^ at 0.1 C and 428 mA h g^−1^ at 8 C after 700 cycles.

Previously, the upshift of the d‐band center was considered to improve the catalytic reaction kinetics. However, optimized engineering for proper binding energies is required referring to Sabatier's principles^[^ ^95^, ^113^, ^114^ ^]^ because excessive strong adsorption causes the reverse effect. Transition metal sulfides generally have moderate electrical conductivity; however, their low adsorption energy toward LiPS poses challenges for their use as catalysts in LSBs.^[^ ^115^, ^116^ ^]^ Shen et al. demonstrated that doping ZnS with TMs (Mn, Fe, Co, Ni, or Cu) resulted in a volcano‐shaped relationship between the LiPS adsorption strength and catalytic activity.^[^ ^54^ ^]^ DFT calculations showed that Mn doping yielded the highest binding energy with LiPS, followed by a sequential decrease, with Cu exhibiting the weakest adsorption. This trend is related to a shift in the d‐band center, which affects the electron occupation of the antibonding sites. To establish a correlation between adsorption strength and reaction kinetics in the volcano relationship, they simplified the conversion steps of Li_2_S_4_ and determined the RDS: the adsorption of Li_2_S_4_ followed by activation/dissociation (Li_2_S_4_* + 2Li^+^ + 2e^−^ → 2Li_2_S_2_*, R2) or desorption (2Li_2_S_2_* → 2Li_2_S_2_ + 2∗, R3). Strong adsorption leads to the RDS in R3, whereas weak adsorption causes the RDS in R2. The highest catalytic activity was observed for Co_0.125_Zn_0.875_S, which exhibited balanced reaction rates in both steps. The optimal catalyst, Co_0.125_Zn_0.875_S, exhibited an extremely low capacity decay of 0.033% over 1500 cycles at 2 C.

As mentioned previously, heterostructures are a promising strategy for tuning electronic structures. Guo et al. fabricated heterostructured Co_9_S_8_@MoS_2_ to achieve proper binding energies toward LiPS.^[^ ^95^ ^]^ A low‐energy d‐band center for MoS_2_ (−2.88 eV) results in weak LiPS adsorption, whereas a d‐band center energy that is too high for Co_9_S_8_ (−1.77 eV) induces surface passivation. However, Co_9_S_8_@MoS_2_, with a value (−2.13 eV) in between those of MoS_2_ and Co_9_S_8_, exhibits accelerated conversion kinetics. With the optimal d‐band center and built‐in electric fields (BIEFs) within the heterointerface, the catalytic effect of Co_9_S_8_@MoS_2_ was significantly improved (Figure 4c–e). Likewise, Zhao et al. synthesized NbN‐NbC heterostructured catalysts, in which highly active facets are stabilized and electron delocalization occurs by coherent nano‐heterocrystal ensembles (CNEs) concept.^[^ ^117^ ^]^ DOSs calculation revealed that the d‐band center of NbN‐NbC {200} facet is upshifted toward *E*_F,_ and that of NbN‐NbC {111} facet is located between NbN and NbC. Not too strong binding, but a proper band center shift enhances catalyst adsorption and ensures sustained catalytic performance. Moreover, electron delocalization at the NbN–NbC interfaces promotes fast charge transfer. Combined with fast charge transfer and high binding energy, Li–S pouch cells with NbN–NbC‐coated separator delivered high energy density of 357 W h kg^−1^.

Metal‐organic frameworks (MOFs), in which metal ions and organic ligands are connected, are widely used as catalysts in electrochemical reactions because of their high surface area, porosity, and tunability, which can be achieved by controlling the metal center and ligand.^[^ ^118^, ^119^, ^120^ ^]^ MOFs typically exhibit low electrical conductivity due to their organic‐metal coordination bonding.^[^ ^121^ ^]^ Therefore, enhancing electron transport is essential not only to adsorb LiPSs but also to facilitate their conversion reactions. The electronic structure of MOFs can be tuned by modifying the type of central metal,^[^ ^122^ ^]^ introducing defects,^[^ ^123^, ^124^ ^]^ or functionalizing the ligands.^[^ ^125^ ^]^ As demonstrated in many previous cases, theoretical calculations are a powerful tool to support specific phenomena. Therefore, prioritizing theoretical calculations to screen materials can be an effective way to reduce experimental efforts. Li et al. reported bidirectional sulfur redox MOFs (M‐BTTC, where M = Mn^2+^, Cu^2+^, or Co^2+^), based on theoretical calculations that predicted the orbital hybridization between transition metal sites and sulfur.^[^ ^126^ ^]^ Their study focused on selecting the optimal metal center among the three ions to achieve a favorable electronic structure when coordinated with the organic ligand benzo[1,2;3,4;5,6]‐tris(thiophene‐2‐carboxylic acid) (H_3_BTTC). According to pDOS of different M‐BTTCs, Co‐BTTC showed the highest d‐band center relative to *E*_F_. As a verification of the theoretical results, they fabricated the M‐BTTC‐modified separators and the Li‐S cells with Co‐BTTC‐modified separators delivered the highest electrochemical performance.

#### 2.1.2 p‐Band Center

Furthermore, owing to their broad electronic structure and lower energy level, the p‐orbital should be considered when the valence electrons of the heterogeneous catalysts are not occupied in the d‐orbitals (e.g., N, O, and Sn) or the electron energy level of the p‐orbitals is upshifted enough to contribute to the valence band. In these cases, the position of the p‐band center relative to the *E*_F_ significantly affects the binding strength between the catalyst and reactants. Therefore, tuning the p‐band center of catalytic materials can regulate their interactions with the reactants, thereby promoting efficient catalysis.

Carbon is one of the promising host candidates due to its lightweight, electrochemical stability, and high porosity with a large surface area, providing enough space to hold sulfur species.^[^ ^127^, ^128^ ^]^ In addition, versatile shape engineering and heteroatom doping in carbon materials induce the accumulation of local charge density and function as catalytic active sites, further improving the sulfur utilization and conservation. Through anion doping in the carbon matrix, it can effectively catalyze the sulfur species. Similar to the d‐band center, Peng et al. demonstrated that the p‐band center of heteroatoms doped in carbon matrix at edge sites can also serve as a descriptor for catalytic activity.^[^ ^129^ ^]^ They synthesized nitrogen and sulfur co‐doped holey graphene framework (N, S‐HGF) electrocatalyst and compared the catalytic effects and p‐band centers of N‐doped (N‐HGF), S‐doped (S‐HGF), and undoped holey graphene framework (HGF). By comparing the activation energies of the SRR, they confirmed that the transition from LiPSs to insoluble Li_2_S_2_/Li_2_S acts as RDS. They identified a volcano‐shaped correlation between the adsorption energy of LiS* formed during the Li_2_S_2_ to Li_2_S transition in SRR and the overpotential. Additionally, they confirmed that the p‐band center is related to the adsorption energy of LiS*, demonstrating its role as a descriptor. N,S‐HGF, with an intermediate p‐band, exhibited high capacities of 1390 and 577 mA h g^−1^ at 0.1 C and 2 C, respectively, under a high areal loading of 4 mg cm^−2^.

Carbon framework not only provides pathways for electron transport to facilitate electrochemical reactions but also offers high stability, which helps mitigate the sulfidation‐related degradation of catalysts in LSBs.^[^ ^130^, ^131^ ^]^ Without heteroatom doping on carbon, Jiang et al. showed that defective carbon has catalytic activity for LiPSs.^[^ ^132^ ^]^ They fabricated defect‐rich carbon nanotubes (CNTs) induced by the reaction of potassium metal intercalated CNT, water, and ethanol. It is known that when defects are generated, electron redistribution around defect sites can be induced and partially positively charged carbon atoms appear.^[^ ^133^ ^]^ With theoretical model of CNTs with different chiral indices, armchair, and defects, they figured out that defective carbon induces the p‐band center upshift according to DFT calculations. Higher capacity retention and rate performance were achieved with defective carbon. Similarly, Zhang et al. reported that defective carbon sites of CNTs were beneficial to LiPSs adsorption and lowered the Li_2_S delithiation energy barrier.^[^ ^134^ ^]^ Additionally, the localized charges of heteroatoms of carbon layers can promote catalytic activity. In 2025, Kim et al. reported a protective catalytic layer (M‐PCL, M = Fe, Co, and Ni), which consisted of a few layers of nitrogen‐doped carbon wrapping different metal cores on CNTs.^[^ ^135^ ^]^ The PCL layer not only prevented the sulfidation of core metal by LiPSs but also provided tuned catalytic activity through interaction with metal cores. Through DFT calculation, they found that the electronic structure of catalytically active pyridinic N sites was affected by the electron‐donating property of central metal species. Co‐PCL, which had appropriate electron donation to the N sites from the Co metal, resulting in an optimized p‐band center, showed the highest catalytic activity. As a result, 1 A h Li–S pouch cell with Co‐PCL delivered a high energy density of 507 W h kg^−1^.

**Figure 5:**
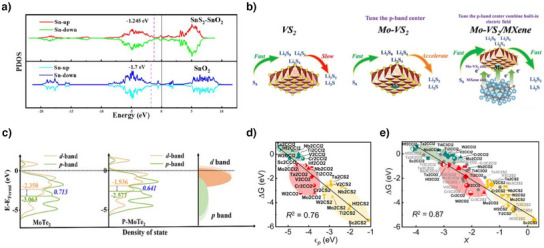
a) pDOS of SnS_2_–SnO_2_ and SnO_2_. Copyright 2024 Elsevier. b) LiPS conversion characteristics with VS_2_, Mo–VS_2_, and Mo–VS_2_/MXene. Copyright 2024 Wiley‐VCH. c) DOS of p‐band, d‐band, and Δband (d–p) center of MoTe_2_ and P‐MoTe_2_. Copyright 2024 Elsevier. d) The relationship between Δ*G* and the surface p‐band center (*ε*_p_). e) The relationship between Δ*G* and the descriptor *X*. *X* is a sum of electronegativity (*χ*_m_) and *ε*_p_. Copyright 2023 American Chemical Society.

Liu et al. introduced a SnS_2_–SnO_2_ heterostructure from SnO_2_ to raise the p‐band center of Sn because the empty p orbitals act as catalytically active centers in elements with d‐orbitals that are full of electrons.^[^ ^52^ ^]^ They verified that the p‐band center of SnS_2_–SnO_2_ (−1.245 eV) was closer to the *E*_F_ compared to SnO_2_ (−1.7 eV) through the pDOS of Sn 3p, as shown in Figure 5a. The upshift of the p‐band center facilitated the adsorption, capture, and conversion processes, ultimately enhancing the catalytic activity in both the SRR and SER.

Wang et al. synthesized Mo‐doped VS_2_ on MXene (Mo‐VS_2_/MXene) via a hydrothermal reaction and investigated the effect of doping on the p‐band center of S in VS_2_.^[^ ^67^ ^]^ Due to the low adsorption strength of VS_2_ toward LiPS, VS_2_ exhibited low catalytic activity. However, Mo doping increased the p‐band center of sulfur in VS_2_, resulting in enhanced interactions with LiPSs. Additionally, the BIEF effect at the interface between MXene and Mo‐VS_2_ facilitated electron transfer. The synergy between the enhanced adsorption with LiPSs and facilitated electron transfer by tuning the p‐band center and BIEF effect, respectively, led to effective catalysis in both the SRR and SER (Figure 5b). Consequently, Mo‐VS_2_/MXene exhibited a high initial capacity of 6.44 mA h cm^−2^ and maintained 5.89 mA h cm^−2^ after 61 cycles at 0.1 C under a high sulfur loading of 5.4 mg cm^−2^ and lean electrolyte condition of 4.5 µL mg^−1^ due to its high catalytic activity. Although valence electrons occupy the d‐orbitals in vanadium, the relative position of the p‐band center to *E*_F_ can be an indicator of the effectiveness of catalysis by the improved influence of the p‐orbital through the upshift of its electron energy level.

While fine‐tuning the interaction between the catalysts and reactants through modulation of the d‐ and p‐band centers is an important factor for effective catalysis, facile electron transfer is also critical. Efficient charge transfer throughout the catalyst can be achieved by the control of the difference between the d‐ and p‐band centers (Δband (d–p) center), as a smaller Δband (d–p) center facilitates smooth charge transfer for the conversion process.^[^ ^136^, ^137^ ^]^ Guo et al. reported that P‐doping of MoTe_2_ on carbon cloth (P‐MoTe_2_/CC) moderately lifted the d‐band center of the Mo atom, as P exhibits relatively high electronegativity.^[^ ^51^ ^]^ However, in the case of MoP, in which Te is completely replaced by P, the d‐band center of Mo increased excessively, resulting in an excessively high binding strength with LiPSs, hindering catalysis by inhibiting LiPS migration. Additionally, partial replacement of Te by P to MoTe_2_ (P‐MoTe_2_) raised not only the d‐band center but also p‐band center. Due to greater up‐shift of p‐band center toward *E*_F_, the Δband (d–p) center was decreased (Figure 5c). Besides, the density of states of d‐ and p‐bands near *E*_F_ are comparable so that the effect of p‐orbital should not be neglected. As a result, a smaller Δband (d–p) center difference results in enhanced orbital hybridization, which accelerates electron transfer and thereby promotes bidirectional conversion reactions between S_6_^2^^−^ and S^2−^ at the interface. As a result, P‐MoTe_2_/CC achieved a high energy density of 350 W h kg^−1^ at 0.5 C. This achievement is attributed to the design of a bidirectional catalyst which reduces the activation barrier of the RDS in the SRR (Li_2_S_2_ to Li_2_S) and SER (delithiation; Li_2_S to LiS + Li) by considering not only the band center but also the Δband (d–p) center. This enabled effective adsorption and conversion processes even under a lean electrolyte of 4.3 µL mg^−1^ when the accessibility of LiPS at the three‐phase interface was reduced due to the viscous electrolyte environment.

In addition, facile electron transfer in a catalyst can be induced through heterostructure engineering. Zhao et al. synthesized pure FeP, Fe_2_P, and FeP/Fe_2_P heterostructured nanoparticles by controlling the heat treatment temperature.^[^ ^106^ ^]^ The FeP/Fe_2_P heterostructure generates an internal electric field at the interface through the Mott–Schottky effect, facilitating charge transfer. To adjust the electronic structure of the interface of this material, it was modeled as FeP_1_ *_–x_* (0 < *x* < 0.5). The FeP_1_ *_–x_* heterostructure exhibited higher catalytic activity than that of pure FeP and Fe_2_P materials. This enhancement can be attributed to not only efficient electron transfer at the interface but also moderate affinity with LiPS, enabling facile adsorption–diffusion–conversion. In addition, there is a small difference between the d‐band center of Fe 3d and the p‐band center of P 2p, which leads to strong interactions between Fe and P, thereby improving the charge transfer and stability of the catalyst.

Electronegativity also significantly influences catalytic performance. MXene is a good catalyst in LSBs owing to its high electrical conductivity and 2D structure. However, identifying the optimal MXene structures depending on the type of transition metal and terminal groups is challenging. Fang et al. proposed a model to predict the SRR activity of MXene based on the Gibbs free energy of the RDS (Li_2_S_2_ → Li_2_S).^[^ ^138^ ^]^ Theoretical calculations showed that surface p‐orbital hybridization and subsurface charge transfer play significant roles in the adsorption of Li_2_S_2_ and Li_2_S. Unlike other metal compounds where the p‐ and d‐band centers influence reactivity, they suggested that for MXene, the descriptor (*X* = *ε*_p_ + *χ*_m_) is represented by the sum of the p‐band center (*ε*_p_) and the electronegativity of the metal layer (*χ*_m_), excluding the subsurface d‐electronic coupling effect. DFT calculations were used to analyze the coefficient of determination (*R* ^2^) values and assess the extent to which the variables explained each other. The *R* ^2^ value of 0.87 between Δ*G*_min_ and *X* = *ε*_p_ + *χ*_m_ was higher than that of 0.76 between Δ*G*_min_ and ε_p_, suggesting that not only the sum of the p‐band center but also the electronegativity of the metal layer significantly influenced the SRR (Figure 5d,e). As a result, they identified Ti_2_CS_2_, Mo_2_CS_2_, and W_2_CS_2_ as the optimal MXenes for promoting the SRR in LSBs based on their screening model. Oxides are well known for their strong affinity for LiPS, resulting in the passivation of the catalyst surface.^[^ ^139^ ^]^ Zhang et al. synthesized TiO_2_ nanoparticles on the halloysite (TiO_2_‐Hal) to modify the p‐band centers of the oxygen sites.^[^ ^56^ ^]^ The low electronegativity of Ti causes electron transfer to O atoms and downshifts the p‐band center from −2.04 to −2.32 eV. Based on the DFT calculations, TiO_2_‐Hal enhanced the desorption of LiPS. Due to the optimized p‐band center, the TiO_2_‐Hal‐embedded cathode delivered a high initial capacity of 1199.1 mA h g^−1^ and cycle stability (87.2% capacity retention) over 200 cycles.

Although the d‐ and p‐band centers of catalysts, calculated based on pDOS, have proven to be effective descriptors for predicting and explaining catalytic activity in LSBs, they possess an inherent limitation in that they do not account for the spatial orientation of LiPS adsorption. For example, the five d orbitals (d*_xy_*, d*_yz_*, d*_xz_*, d*_x_*_2_ *_–y_*_2_, and d*_z_*_2_) possess distinct spatial symmetries, and the extent to which they overlap with the orbitals of LiPS varies depending on the adsorption geometry on the catalyst surface. Therefore, incorporating orbital‐specific weighting based on the extent of spatial overlap between LiPS and each orbital of the catalyst upon adsorption could serve as a more accurate descriptor of catalytic activity.

#### 2.1.3 Band Gap

Efficient electrochemical redox of sulfur species requires strong adsorption, rapid mass transport, effective Li⁺ ion diffusion, and fast charge transfer.^[^ ^140^ ^]^ In addition, during the electrochemical redox process, electrons are transferred from active materials and migrate to the other electrode; thus, efficient electronic conduction plays a crucial role in catalytic activity. Despite catalysts with fast charge transfer, limited electronic conduction throughout the electrode can still hinder overall catalytic performance.^[^ ^85^ ^]^ Band gap, defined as the energy difference between the valence band maximum (VBM) and the conduction band minimum (CBM), influences the electron mobility and thus affects the material's electrical conductivity. A smaller band gap accelerates the electron transportation. Especially, CNTs and graphene are widely used themselves or as supports of catalysts, providing electrical pathways because of their sp^2^ hybridized structure, in which delocalized electrons are beneficial to high electrical conductivity.^[^ ^89^, ^141^ ^]^

**Figure 6:**
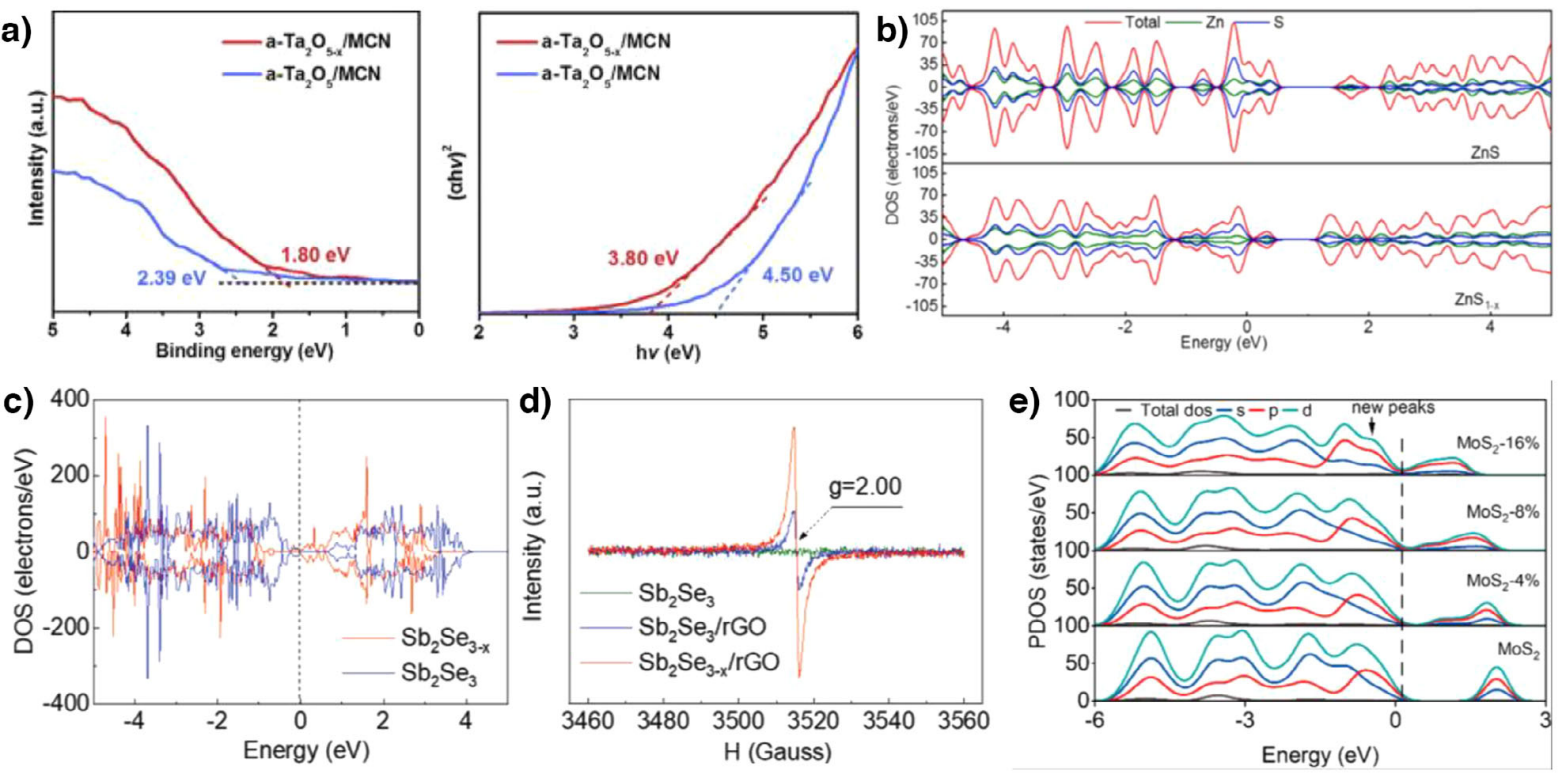
a) XPS valence band spectra and Kubelka–Munk plot of a‐Ta_2_O_5_/MCN and a‐Ta_2_O_5_ *_–x_*/MCN. Copyright 2020 Cell Press. b) DOS of ZnS and ZnS_1–_ *_x_*. Copyright 2021 Elsevier. c) DOS of Sb_2_Se_3_ and Sb_2_Se_3–_ *_x_*. d) EPR spectra of Sb_2_Se_3_, Sb_2_Se_3_/rGO and Sb_2_Se_3−_ *_x_*/rGO. Copyright 2019 Wiley‐VCH. e) pDOS of Li_2_S_4_ on MoS_2_ with different defect level. Copyright 2023 American Chemical Society.

In general, only using low electrically conductive catalysts such as metal oxides or sulfides limits the catalytic activity. To compensate for the low electrical conductivity, carbon composites are widely used. General metal oxide compounds have high affinity to LiPSs but their intrinsic low electrical conductivity interrupts catalytic activity. Defect on catalysts is an effective strategy to induce fast electron migration. In 2020, Zhang et al. used amorphous oxygen vacant a‐Ta_2_O_5_ *_–x_* nanoclusters confined in micropores of carbon nanospheres (a‐Ta_2_O_5_ *_–x_*/MCN).^[^ ^142^ ^]^ Compared to low electrical conductivity of both amorphous Ta_2_O_5_ (a‐Ta_2_O_5_, 3.4 × 10^−7^ S m^−1^) and crystalline Ta_2_O_5_ (T‐Ta_2_O_5_, 3.4 × 10^−7^ S m^−1^), that of a‐Ta_2_O_5_ *_–x_* was significantly increased almost 10^5^ times. As shown in Figure 6a, through XPS VB spectra and Kubelka–Munk plot, band diagram of Ta_2_O_5_ catalysts was obtained, and they found that the band gap of a‐Ta_2_O_5_ *_–x_*/MCN was narrowed. In addition, electron paramagnetic resonance (EPR) spectroscopy of a‐Ta_2_O_5_ *_–x_*/MCN shows at *g* = 2.003, which indicates oxygen defect formation. With a‐Ta_2_O_5_ *_–x_*/MCN, Ta‐based sulfur electrode maintained high capacity over 4 mA h cm^−2^ under high sulfur loading of 5.6 mg cm^−2^ and low *E*/*S* ratio (3.6 µL mg^−1^) condition.

Not only oxygen vacancy, but other anions such as nitrogen, sulfur, phosphorus, selenium, and tellurium vacancy sites also accelerate the electron conduction.^[^ ^143^ ^]^ For example, Wang et al. synthesized sulfur‐deficient ZnS nanotubes on carbon cloth (ZnS_1_ *_–x_* CC) for sulfur electrode.^[^ ^63^ ^]^ They found that sulfur defect facilitates the LiPSs adsorption, Li_2_S delithiation, and Li^+^ ion diffusion. Additionally, the narrowed band gap of ZnS_1_ *_–x_* (0.5 eV) compared to that of ZnS (0.75 eV) enhances electrical conductivity (Figure 6b). Similarly, Tian et al. fabricated selenium deficient Sb_2_Se_3_ wrapped by rGO catalysts (Sb_2_Se_3_ *_–x_*/rGO) through thermal shock method at 600 °C Ar atmosphere.^[^ ^61^ ^]^ Unlike clear band gap of Sb_2_Se_3_, as shown in Figure 6c, newly formed defect level of Sb_2_Se_3_ *_–x_* below conduction band facilitate the electron conduction from valence band to conduction band. Also, Se defect sites induced EPR signal at *g* = 2.00 (Figure 6d). Therefore, the electrical conductivity of Sb_2_Se_3_ *_–x_*/rGO increased to 6.25 × 10^4^ S m^−1^, which is 10 times higher than that of nondefective Sb_2_Se_3_/rGO. With enhanced electrical conductivity, LSBs with Sb_2_Se_3_ *_–x_*/rGO‐modified separator delivered a high capacity of 1249 mA h g^−1^ at 0.2 C and 787 mA h g^−1^ at 8.0 C.

Although enhanced electrical conductivity of catalysts through defect‐induced band structure modulation is often emphasized, it should be noted that this is not the sole reason for the improved catalytic activity. Rather, a comprehensive understanding of how defect formation influences various aspects of catalytic performance is necessary. Guo et al. showed the importance of proper amount of defect sites using MoS_2_.^[^ ^144^ ^]^ As the reduction temperature of pristine MoS_2_ increased from 300 to 700 °C under H_2_/Ar gas, corresponding to MoS_2_‐3 to MoS_2_‐7, they could regulate the amount of defect sites on MoS_2_. According to the calculated pDOS of MoS_2_ with 0–16% defect levels, the band gap was narrowed as defect levels increased, which accelerates the electron migration (Figure 6e). Interestingly, MoS_2_‐5 delivered the highest electrochemical performance even compared to MoS_2_‐6 and MoS_2_‐7, which have narrower band gaps. According to the binding energy calculation of LiPSs, the binding energies of MoS_2_‐6 and MoS_2_‐7 toward Li_2_S_6_ and Li_2_S were too high to facilitate the desorption, disturbing catalytic activity. Therefore, adequate amount of defect formation should be achieved to improve overall catalytic activity.

In addition to enhancing the intrinsic electrical conductivity of the catalyst through band gap narrowing, effective catalytic activity also requires efficient charge transfer between the catalyst and the adsorbed species. Therefore, considering the relative alignment between the CBM and VBM of the catalyst and the LUMO and HOMO levels of LiPSs can be critical for accurately predicting the electrical conductivity and activity of catalysts.

### 2.2 Electronic Properties

In heterogeneous catalysts, electronic structure parameters, such as the d‐band center and p‐band center, play a critical role in enhancing catalytic activity by modulating electronic interactions with both catalysts and reactants. In addition to these structural factors, electronic properties such as the spin state and Lewis acidity/basicity also significantly influence the mutual interactions between the catalysts and reactants. These properties are therefore essential considerations in the rational design of highly active catalysts for LSBs. Furthermore, in the case of homogeneous catalysts such as RMs, the redox potential, as an electronic property, determines the electronic interactions with solution‐phase reactants. By modulating these electronic properties, it becomes possible to tailor the binding strength and charge transfer characteristics, ultimately enabling effective catalysis in both the SRR and SER.

#### 2.2.1 Spin State

The degenerate d‐orbitals of TMs (d*_xy_*, d*_yz_*, d*_xz_*, d*_x_*_2_ *_–y_*_2_, and d*_z_*_2_) undergo energy splitting owing to repulsion from the electrons of the surrounding ligands. The properties of the metal ion and the ligand field cause variations in the electrostatic interactions between the ligand electrons and each d‐orbital, influencing the energy levels of the split d‐orbitals.^[^ ^145^ ^]^ Consequently, the arrangement of electrons in the d‐orbitals depends on the degree of energy splitting and the pairing energy, leading to stable configurations that affect the electronic structure. When the energy splitting is small, the high‐spin configurations become stabilized, causing electrons to occupy higher energy levels and resulting in an upshift of the d‐band center.^[^ ^146^ ^]^ In addition, unpaired electrons enhance electron transfer. Therefore, adjusting the spin state can enhance the activity of the heterogeneous catalyst.

**Figure 7:**
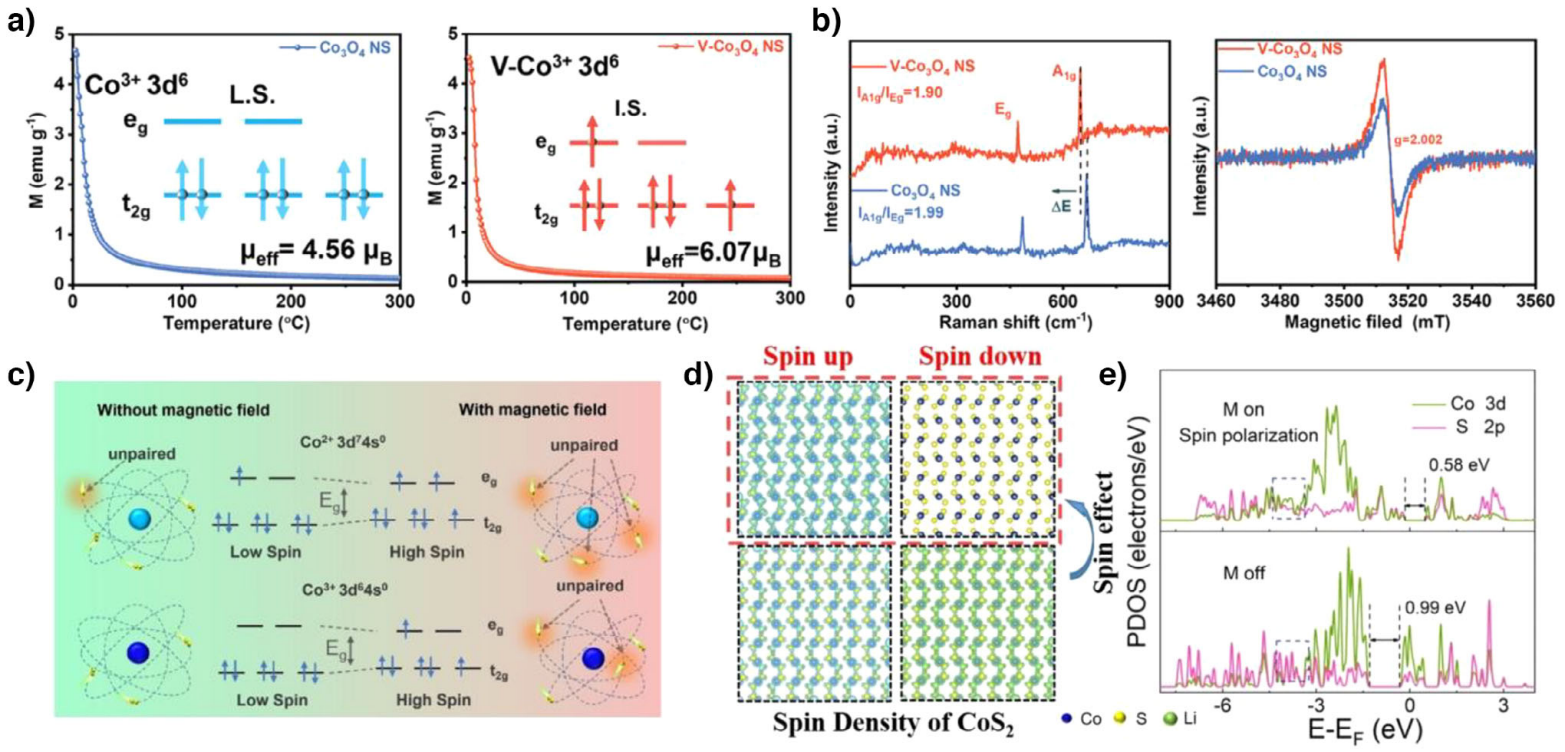
a) Low spin state of Co_3_O_4_ and intermediate spin state of V‐Co_3_O_4_ and their magnetic susceptibility. b) Effects of the spin state of Co^3+^ on the formation of the bonding orbital with LiS*. Copyright 2024 Wiley‐VCH. c) Spin state modulation of Co^2+^ and Co^3+^ in CNF/CoS*_x_* under a magnetic field. d) The difference in spin density of CoS_2_ under an external magnetic field. e) Increase of overlap between Co 3d and S 2p by spin polarization. Copyright 2022 Wiley‐VCH.

Zhou et al. used V‐doped Co_3_O_4_ to alter the spin state of Co^3+^.^[^ ^57^ ^]^ As shown in Figure 7a, the crystal field splitting energy causes Co^3+^ to adopt a low spin state due to the strong ligand field of O^2−^. Raman spectroscopy and X‐ray photoelectron spectroscopy demonstrated that V primarily interacted with Co^3+^ rather than Co^2+^. Using EPR, they confirmed an increase in the number of unpaired electrons, indicating that V‐doping modified the spin‐state configuration of Co^3+^, enhancing its spin state (Figure 7b). The altered electronic structure of Co^3+^ ultimately increased the bond order with LiS* from 0.5 to 1.5 during molecular orbital formation, improving its adsorption strength. Furthermore, the improved adsorption capability due to the spin state change of Co^3^⁺ induced by V‐doping contributed to an increase in the bond length of the S─S bond in Li_2_S_4_ and the Li─S bond in Li_2_S, enabling its effectiveness as a bidirectional catalyst for both the SRR and SER. As a result, the Li–S cell, utilizing a separator modified with V‐Co_3_O_4_, exhibited a high reversible capacity of 15.37 mA h cm^−2^ and cycle stability, retaining 89% of its capacity over 100 cycles.

The magnetic field influences the magnetic moment generated by an electron spin, causing the spins to align either parallel or antiparallel to the magnetic field.^[^ ^147^, ^148^ ^]^ Consequently, an external magnetic field can affect the spin state of the catalyst, resulting in a change in the catalytic properties. Zhang et al. synthesized CNF/CoS*_x_* and reported that its enhanced catalytic activity was due to changes in the spin state of Co^2+^ under an external magnetic field (Figure 7c).^[^ ^58^ ^]^ In the absence of a magnetic field, both Co^2+^ and Co^3+^ exhibit a low‐spin state; however, under the influence of a magnetic field, spin alignment causes a shift to a high‐spin state. As shown in Figure 7d,e, the spin is aligned with the external magnetic field. This enhances the hybridization between the Co‐3d and S‐2p orbitals, strengthening the interaction with the polysulfides (PSs). Enhanced by the magnetic field effect, CNF/CoS*_x_*/S exhibited a high capacity (1464.7 mA h g^−1^) at 0.1 C and impressive rate capability of 793.7 mA h g^−1^ at 4 C. Furthermore, Li et al. demonstrated that the electron configuration of the split *e*_g_ and *t*_2g_ orbitals in TM catalysts is a key factor determining the catalysis of LiPSs.^[^ ^141^ ^]^ The molecular orbitals formed between the metal and LiPS were influenced by the filling of the antibonding orbitals. Electrons in the *e*_g_ orbitals can stabilize the PS structure by maintaining the S─S bond to prevent PS degradation, whereas electrons in the *t*_2g_ orbitals may weaken the interactions between the metal and PSs. Therefore, a higher *e*_g_/*t*_2g_ ratio is associated with enhanced PS conversion reactions. As a result, catalytic materials of a CoZn cluster with carbon that have the highest *e*_g_/*t*_2g_ value exhibited an excellent rate performance, with a high initial power (26 120 W h k^−1^ s^−1^) at 8 C over 1000 cycles.

#### 2.2.2 Lewis Acidity and Basicity

**Figure 8:**
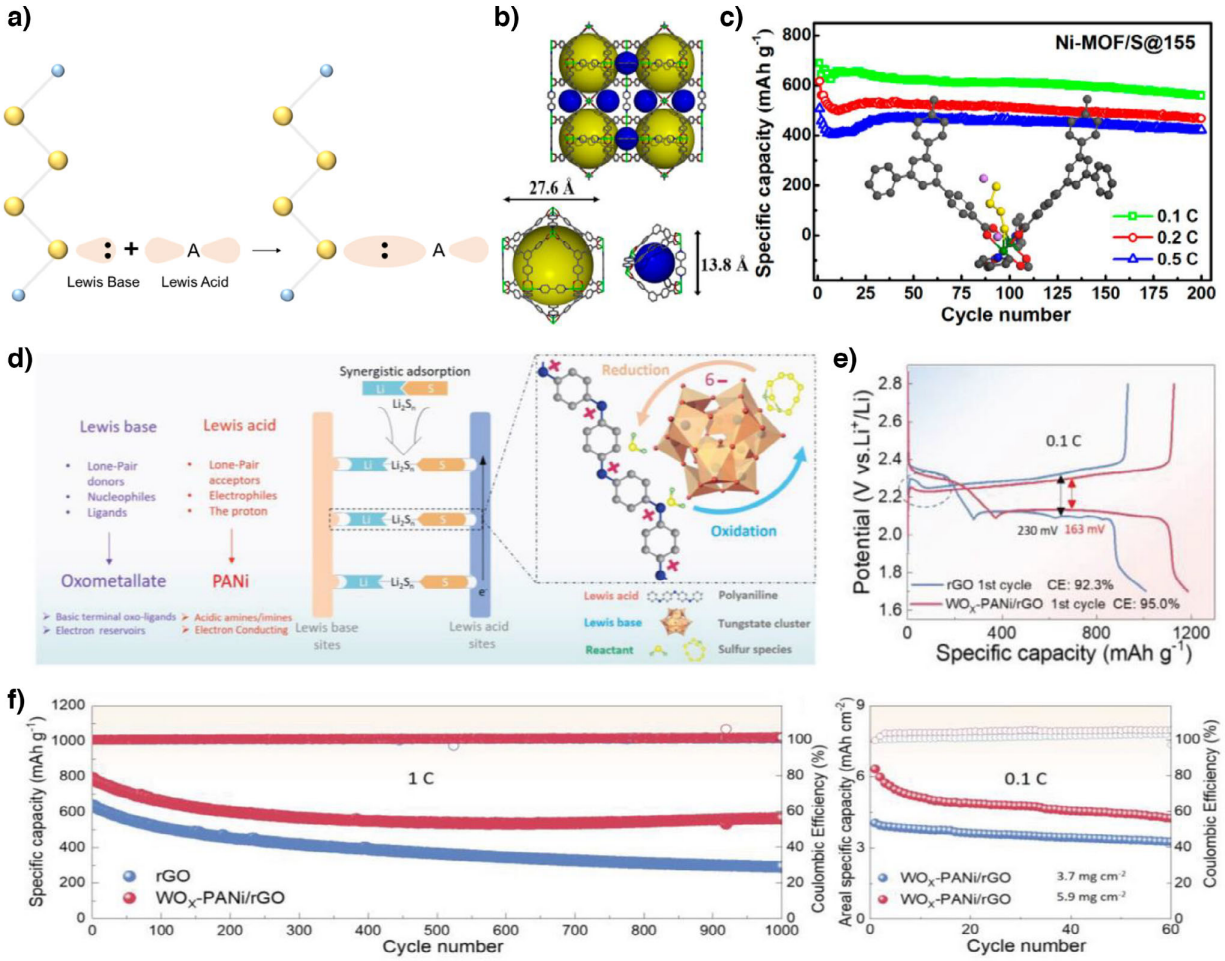
a) Schematic concept of Lewis acid and base. b) The crystal structure of Ni_6_(BTB)_4_(BP)_3_ (BTB = benzene‐1,3,5‐tribenzoate and BP = 4,4′‐bipyridyl). c) Lewis acid and base interaction of Li_2_S_4_ and Ni‐MOF with its cycle stability of Ni‐MOF/S@155 cathode. Copyright 2014 American Chemical Society. d) Conceptual strategy of collective Lewis acid and base sites for bidirectional catalyst (WO*_x_*‐PANi/rGO). e) Voltage profile at 0.1 C for comparing polarization of rGO and WO*_x_*‐PANi/rGO. f) Galvanostatic long‐term cyclability test of LSBs with rGO and WO*_x_*‐PANi/rGO. Copyright 2024 Wiley‐VCH.

Lewis acid–base interactions can be utilized to fine‐tune electronic interactions between catalyst and adsorbate to achieve high catalytic activity. The Lewis acid–base theory describes the interaction between acids that can accept electron pairs and bases that can donate them (Figure 8a).^[^ ^149^ ^]^ Control of Lewis acid–base interactions can be achieved through a heteroatom doping strategy because the lone pair electrons of the dopants act as Lewis bases, and the terminal Li atoms in LiPSs act as Lewis acid sites. Hou et al. reported the effect of heteroatom dopants on the adsorption of LiPS onto a carbon matrix.^[^ ^150^ ^]^ They discovered that N and O had more favorable LiPS anchoring energies than B, F, S, P, and Cl and that lone pair electrons should exist for the Lewis acid interactions of the Li‐doping atoms. Additionally, they suggested the importance of electronegativity, radius, stability, and electronic structure in heteroatom doping strategies. The hard and soft acid and base (HSAB) theory is widely used to predict the stability and reactivity of Lewis acid and base materials.^[^ ^151^ ^]^ Sulfur in LiPS is a soft Lewis base owing to its high electron density, large atomic size, and highly deformable electron cloud with high polarizability.^[^ ^152^, ^153^ ^]^ According to HSAB theory, LiPS easily interacts with soft Lewis acids. The metal ions in MOFs can act as Lewis acids by accepting electrons, which makes them suitable catalysts because of their numerous active sites. Zheng et al. used a Ni‐based MOF, Ni_6_(BTB)_4_(BP)_3_ (BTB = benzene‐1,3,5‐tribenzoate; BP = 4,4′‐bipyridyl), as the host and catalyst (Figure 8b).^[^ ^59^ ^]^ Ni^2+^, with its soft Lewis acid properties, interacted strongly with PSs, and the Ni‐MOF/S@155 cathode exhibited high cycle stability, as shown in Figure 8c.

Because of the complexity and opposing reaction pathways of the SRR and SER, it is challenging to design a catalyst that simultaneously promotes both reactions in LSBs. Nevertheless, Lin et al. designed a bifunctional catalyst for both charge and discharge processes through Lewis acids, accelerating the SER by accepting electrons from PS and Lewis base sites and promoting the SRR by donating electrons to Li^+^.^[^ ^60^ ^]^ They used polyaniline (PANi), a conductive polymer, for Lewis acid sites and synthesized protonated metatungstate anions ([H_2_W_12_O_40_]^6−^), which serve as Lewis base sites, onto the PANi (Figure 8d). The resulting WO*_x_*–PANi catalyst, with both Lewis acid and base sites functioning simultaneously, promoted the SRR and SER more effectively than the individual catalysts. As a result, it exhibited reduced polarization and a low capacity decay of 0.029% over 1000 cycles at 1 C. Furthermore, the WO*_x_*–PANi/rGO‐incorporated cathode cycled stably for more than 60 cycles at a high sulfur loading (5.9 mg cm^−2^) and low *E*/*S* ratio (5.7 µL mg^−1^) (Figure 8e,f).

#### 2.2.3 Redox Potential

RMs that can be solvated in the electrolyte are homogeneous catalytic species that can facilitate a sluggish sulfur conversion reaction. They undergo reversible redox reactions at the electrode surface via electrochemical oxidation or reduction and subsequently diffuse through the electrolyte to chemically oxidize or reduce sulfur species.^[^ ^64^ ^]^ RMs can be solvated in the electrolyte, thereby facilitating the formation of the triple‐phase interface more readily than heterogeneous catalysts. This characteristic makes them particularly effective under lean electrolyte conditions, where high electrolyte viscosity significantly hinders ion and mass transport. The redox potential is an electronic property that reflects a chemical species' tendency to donate or accept electrons during a redox reaction. The RMs with appropriately tuned redox potentials can reactivate dead sulfur or Li_2_S through solution‐mediated electron transfer and promote the 3D growth of Li_2_S by preventing passivation of electrode surface with insulating Li_2_S, thereby enhancing the reversible capacity.

**Figure 9:**
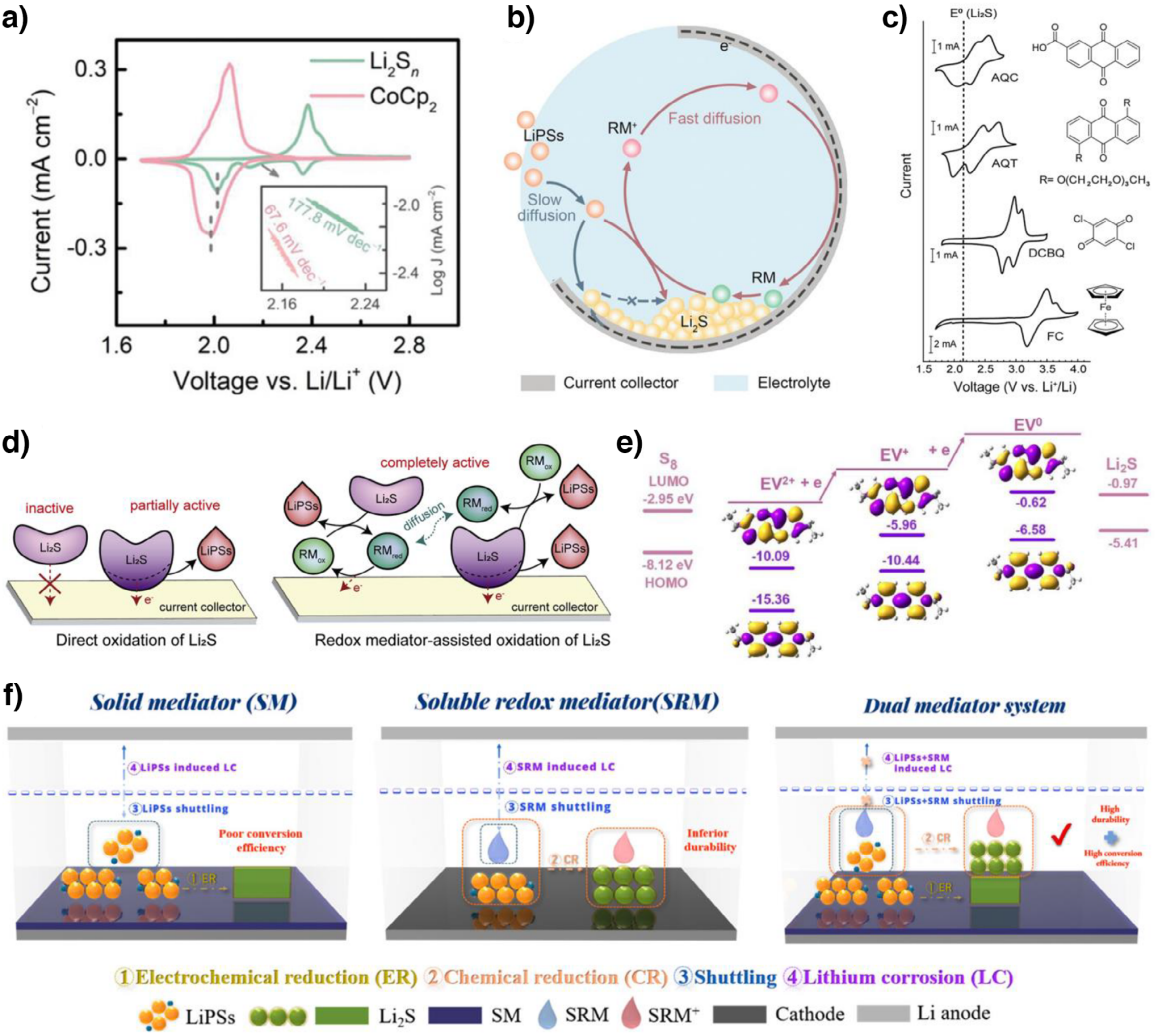
a) Redox potential of cobaltocene (CoCp_2_) compared to Li_2_S conversion reaction. b) A schematic illustration of the Li_2_S conversion reaction mechanism facilitated by the extrinsic redox mediator (RM) CoCp_2_. Copyright 2019 Wiley‐VCH. c) Redox potential of four different RMs analyzed by cyclic voltammetry. d) Schematic illustration of the difference in Li_2_S oxidation behavior with and without the RM. Copyright 2019 Elsevier. e) Frontier molecular orbital of different oxidation states of EV(ClO_4_)_2_ compared to those of Li_2_S and S_8_. Copyright 2021 Elsevier. f) Synergistic effect of solid mediator and soluble RM in LSBs. Copyright 2022 American Chemical Society.

In LSBs, the specific redox potential of RMs determines which reactions it can effectively facilitate. The Li_2_S conversion reaction (Li_2_S_4_ + 6 Li^+^ + 6 e^−^ → 4 Li_2_S) accounts for approximately 75% of the theoretical capacity and proceeds slowly, making its promotion critically important. Moreover, inducing the 3D growth of Li_2_S is also essential for high‐energy‐density LSBs. Zhang's group utilized cobaltocene (CoCp_2_), whose redox potential is 27 mV lower than the Li_2_S conversion reaction (Figure 9a).^[^ ^154^ ^]^ The slightly lower redox potential of RM than that of Li_2_S conversion reaction facilitated the formation reaction of Li_2_S by following equation (CoCp_2_^+^ + Li_2_S*_n_* → CoCp_2_ + Li_2_S) without significant average discharge voltage drop. The enhanced Li_2_S conversion reaction with CoCp_2_ was verified by a higher *Q*_Low_/*Q*_High_ value (4) compared to that without CoCp_2_ (3.7) in the galvanostatic cycling test at 0.05 C. Additionally, CoCp_2_ effectively promoted the 3D growth of Li_2_S. In the absence of CoCp_2_, the growth of Li_2_S proceeded primarily through the mediation of intrinsic RMs such as LiPS, and is confined to the triple‐phase boundary formed by the electrolyte, conductive support, and Li_2_S, resulting in 2D growth. In contrast, the small molecular size of CoCp_2_ enabled its rapid diffusion toward polar Li_2_S nuclei and facilitated the conversion of LiPS into Li_2_S, thereby enabling 3D growth of Li_2_S (Figure 9b). Consequently, the LSBs with CoCp_2_ showed a higher discharge capacity of 1148 mA h g^−1^ and lower voltage polarization of 198 mV at a low *E*/*S* ratio of 4.7 µL mg^−1^ and 0.05 C.

In addition, Bao's group investigated the influence of RM's redox potential on Li_2_S oxidation reaction using four different RMs: 1) anthraquinone‐2‐carboxylic acid (AQC); 2) anthra‐9,10‐quinone (AQT); 3) 2,5‐dichloro‐1,4‐benzoquinone (DCBQ); and 4) ferrocene (FC).^[^ ^155^ ^]^ Li_2_S oxidation reaction, whose redox potential is 2.15 V (vs. Li^+^/Li^0^), showed large overpotential due to the insulating nature of Li_2_S and the difficulty in breaking the strong Li─S bond in Li_2_S. Among them, AQT demonstrated an effective performance in chemically oxidizing Li_2_S without inducing a significant increase in overpotential because of its slightly higher redox potential than Li_2_S oxidation potential (Figure 9c). Because the solubilized oxidized form of AQT chemically oxidized isolated Li_2_S while being reduced itself, and was subsequently electrochemically reoxidized at the electrode surface (Figure 9d), LSBs with AQT exhibited excellent cycling stability, retaining a high reversible capacity of 850 mA h g^−1^ after 500 cycles at 1 C.

The electron‐donating or ‐accepting tendency of RMs can be fundamentally explained by the energy levels of their frontier molecular orbitals (FMOs), namely the highest occupied molecular orbital (HOMO) and the lowest unoccupied molecular orbital (LUMO). The positions of these orbitals determine the redox potential of the RMs, thereby enabling the prediction of their roles in LSBs. Ye et al. introduced ethyl viologen diperchlorate (EV(ClO_4_)_2_) as an RM that scavenges both the dead S and Li_2_S.^[^ ^156^ ^]^ Reactivation of dead S and Li_2_S by EV(ClO_4_)_2_ was elucidated by the HOMO and LUMO levels of its redox states (EV^0^, EV^+^, and EV^2+^) compared to those of S_8_ and Li_2_S (Figure 9e). Since LUMO level of EV^2+^ is lower than HOMO level of Li_2_S and HOMO level of EV^0^ is higher than LUMO level of S_8_, effective reactivation of both isolated S and Li_2_S by EV(ClO_4_)_2_ was enabled. Therefore, the LSB with EV(ClO_4_)_2_ exhibited higher capacity retention of 63% after 100 cycles compared to that without EV(ClO_4_)_2_ under a lean electrolyte condition (4.7 µL mg^−1^), where supersaturation‐induced precipitation of Li_2_S and sulfur, coupled with restricted mass transport, critically promotes the accumulation of electrochemically inactive Li_2_S/S species on the electrode surface.^[^ ^157^ ^]^

However, the shuttling behavior of solvated RMs impedes their sustained redox mediation, which in turn impairs the cycling stability of LSBs. Introducing heterogeneous catalysts capable of strong interactions with RMs has been proposed as a potential strategy to mitigate this issue. Jiao et al. proposed a dual mediator system with a heterogeneous catalyst of CoS_1.097_ nanoparticles in N‐doped porous carbon sheet (CoSNC) and homogeneous catalyst of CoCp_2_.^[^ ^158^ ^]^ When using only heterogeneous catalysts, the sluggish sulfur conversion reaction can be promoted, but the reaction is confined to the limited catalytic surface. As insulating film‐like Li_2_S gradually covers the electrode surface, further catalytic activity is hindered. On the other hand, employing only soluble homogeneous catalysts allows for the chemical reduction of LiPS and facilitates the 3D growth of Li_2_S, enabling high catalytic activity due to the easier formation of the triple‐phase interface. However, the shuttling of soluble RM not only leads to their depletion but also Li corrosion (Figure 9f). In contrast, when both types of catalysts are used together, strong interactions between heterogeneous and homogeneous catalysts suppress the shuttling of the homogeneous catalyst, allowing sustained and effective redox mediation. Consequently, dual mediator system of CoSNC and CoCp_2_ exhibited a low capacity decay ratio of 0.026% per cycle over 1200 cycles at 2 C by their synergistic effect.

## 3 Summary and Outlook

In this review, we introduced the importance of designing optimized catalysts to accelerate the SRR and SER by understanding their electronic structures and properties. In particular, a deep understanding of the interaction between LiPS and the d‐band center of catalytic materials based on their electronic structure and properties is necessary for designing effective bidirectional catalysts.^[^ ^52^ ^]^ Therefore, modulating the interactions between the catalysts and LiPSs is critical for designing catalysts with high activity. The position of the d‐band center relative to the *E*_F_ is an important indicator that assists in predicting the binding strength of catalysts toward LiPSs.^[^ ^159^ ^]^ According to Sabatier's principle, appropriate improvement of the binding strength has been considered a critical factor in achieving efective and durable catalysis.^[^ ^160^ ^]^ The p‐band, which has been considered relatively less than the d‐band, can also play an important role in understanding the interaction between catalysts and LiPSs by modulating their electronic structure.^[^ ^67^ ^]^ In addition, the modification of the electronic properties of catalysts, such as the spin state and Lewis acidity and basicity, is an efficient strategy for tailoring the electronic structure of the catalyst in the desired directions by diverse catalyst design tactics (doping, defect, and heterostructure).^[^ ^60^, ^161^ ^]^

We proposed future‐orienteddesign strategies for catalyst development to construct bidirectional catalysts with optimized activity for high‐energy density and long‐life LSBs. Transition‐metal‐based catalytic materials that can utilize d‐orbitals exhibit high potential for achieving superior catalytic performance owing to the high electron density of the d‐orbital and multiple oxidation states. Therefore, SACs and TMCs based on d‐orbitals are promising candidates as effective bidirectional catalysts in LSBs. As discussed above, fine‐tuning the interactions between the catalysts and LiPSs is the key to facile SRR and SER.^[^ ^20^, ^162^ ^]^ Therefore, the interaction can be modulated through the following approaches, depending on the type of catalytic material. In the case of SACs, their interactions with LiPSs can be controlled by diverse strategies: 1) heteroatom doping to create an asymmetric environment, 2) reorganization of the electronic structure of the metal atom center by modifying the electron transfer properties, and 3) creation of multiple atom sites (e.g., dual‐atom catalysts). In the case of TMCs, their interactions with LiPSs can be modulated through changes in the electronic structures of transition metals and anions by various factors, such as the element type of the anion, defects, doping, and crystal structure. Ultimately, collaboration between computational theory and experiments is essential to precisely predict and verify the electronic structure of catalysts and their interactions with LiPSs, as computational insights guide experimental designs, and experimental results refine theoretical models.

Furthermore, identifying appropriate descriptors based on electronic structure to predict catalytic properties is essential for designing catalysts with higher catalytic performance. Achieving this requires a close collaboration between computational approaches, such as machine learning and artificial intelligence, and experimental studies on LSBs. This is because the complex charge–discharge mechanisms of LSBs and the sulfur conversion reaction are influenced by various factors, including the electronic effects (e.g., band center and spin state) and structural effects (e.g., d‐spacing and crystal plane) of the catalyst. Additionally, in situ and ex situ analyses are essential not only for analyzing the catalytic mechanisms of the developed catalysts but also for understanding catalyst degradation to ensure durability. N, the catalysts can undergo sulfidation during charge/discharge cycles because of LiPS corrosion.^[^ ^56^ ^]^ This phenomenon can change the intended electronic structure of the catalyst, resulting in degradation of the catalytic activity. Therefore, it may be necessary to develop catalysts with protective surfaces or enhanced corrosion resistance to prevent such degradation.

While the electronic perspective we introduced is a major factor in determining the interactions with LiPS, it is important to recognize that several other factors, such as Li^+^ ion diffusion,^[^ ^163^ ^]^ the bond angle of deposited Li_2_S,^[^ ^164^ ^]^ and electron transport^[^ ^165^ ^]^ can also influence the SRR and SER. In addition to modulating the electronic structure of the catalyst, simultaneous consideration of the physical properties of the catalysts, such as the exposure of active sites, sulfur impregnation, electrical conductivity, and Li^+^ ion accessibility, is essential to maximize catalytic performance. In summary, developing highly active heterogeneous catalysts is a complex task that requires a thorough understanding of catalysis, electronic structure, and physical properties of catalytic materials.

In addition to heterogeneous catalysts, soluble RMs have recently emerged as promising homogeneous catalytic species for promoting sulfur conversion reactions through solution‐mediated electron transfer even under lean electrolyte conditions. The redox potential and FMO levels of RMs are key factors influencing their reactivity and selectivity. RMs have been shown to promote 3D growth of Li_2_S and reactivate dead sulfur species, thereby improving reversibility. The design strategies for soluble RMs include the following: 1) Exploration of the redox potential of RMs within the voltage window of LSBs; 2) Consideration of the diffusivity and solubility of RMs to ensure their redox mediation through solution‐mediated charge transfer; 3) Modulation of redox potential and FMO (HOMO/LUMO) levels of RMs affecting reaction selectivity and energy losses in LSBs. Since the redox potentials and FMO levels of RMs are highly influenced by their solvation environment, theoretical calculations should be performed under solvated conditions rather than in vacuum to more accurately reflect their in situ redox behavior; 4) Investigation of RM's reversibility over prolonged cycling. The shuttling behavior and irreversible side reactions of RMs with reactive Li metal were significant challenges in LSBs.

To address these limitations, the development of RMs is considered to benefit from being accompanied by rational electrolyte design. Tuning the dielectric constant of the electrolyte can not only regulate RM solubility but also stabilize their charged redox states, resulting in redox potential modulation. Additionally, the introduction of fluorinated cosolvents as weakly coordinating diluents can be an effective strategy that offers dual benefits: suppressing direct contact between RMs and the Li metal anode and facilitating LiF‐rich SEI formation. These strategies may synergistically mitigate RM shuttling and contribute to the long‐term stability and effectiveness of RMs in LSBs. Ultimately, we believe that a comprehensive understanding of catalysts from an electronic perspective, regardless of their type, is essential to develop highly active catalytic systems and to enable the commercialization of LSBs.

## Conflict of Interests

The authors declare no conflict of interest.

## Data Availability

Data sharing is not applicable to this article as no new data were created or analyzed in this study.
